# A singularity in gender difference in medicine: nervous system and behavior

**DOI:** 10.1265/ehpm.26-00157

**Published:** 2026-10-21

**Authors:** Gaku Ichihara, Akiko Matsumoto, Sahoko Ichihara, Atsuko Ikeda, Yuko Yamano, Yuki Kitamura, Chiharu Nishiyama

**Affiliations:** 1 Faculty of Pharmaceutical Sciences, Tokyo University of Science; 2 Faculty of Medicine, Saga University; 3 Jichi Medical University School of Medicine; 4 Faculty of Health Sciences, Hokkaido University; 5 Faculty of Medicine, Showa Medical University; 6 Faculty of Advanced Engineering, Tokyo University of Science

**Keywords:** Gender, Neurodegenerative disease, Behavior

## Abstract

A symposium entitled “A singularity in gender difference in medicine: nervous system and behavior” was held on 21^st^ March 2025 at the 85^th^ Annual Meeting of the Japan Society for Hygiene in Saitama, Japan. Three speakers presented reviews on gender difference in neurodegenerative disease including Alzheimer disease and actions of sex steroid and regulation of their receptors, and one speaker presented an original study on gender difference in behavior of twins. The symposium concludes that further research is needed to clarify the roles of estrogen and genetic factors to better understand the mechanisms underlying gender differences in neurodegenerative diseases and behavioral problems.

## Introduction

A symposium entitled “A singularity in gender difference in medicine: nervous system and behavior” was held on 21^st^ March 2025 at the 85th Annual Meeting of the Japan Society for Hygiene in Saitama, Japan. The symposium was organized through collaboration among the Japan Society for Hygiene, the Japan Society for Occupational Health, the Japan Society for Neurology, and the Japan Society for Anatomy, and was funded by the Japanese Medical Sciences Federation.

## Gender difference in neurodegenerative diseases

Prof. Toshimasa Yamamoto reviewed gender differences in neurodegenerative diseases. The prevalence of Parkinson’s disease (PD) is 1.57-fold higher in males than in females [1]. The annual incidence rate of PD is 1.59-fold higher incidence in males than in females. However, in Japan and South Korea, both the prevalence and annual incidence of PD are higher in females than in males, suggesting regional differences. In females, there is an association between PD prevalence and decreased estrogen levels, which is related to early menopause or frequent childbirth. Reported gender differences in the prevalence of **dementia with Lewy bodies (DLB)** vary across studies. Female patients with DLB tend to die at an older age and show greater accumulation of tau protein compared with male patients. No clear gender difference in the prevalence of **multiple system atrophy (MSA)** has been reported. Depression is more frequently reported in female patients with MSA, whereas orthostatic hypotension is more frequently reported in male patients. Gender differences in the prevalence of **progressive supranuclear palsy (PSP):** have not been clearly determined. The prevalence of **Alzheimer’s disease (AD)** is reported to be approximately twofold higher in females than in males [2]. Longer lifespan, genetic factors, and sex hormones have been suggested as possible explanations for this difference. Prof. Yamamoto concluded that gender differences may influence neurological symptoms and prognosis in neurodegenerative diseases. Further investigation into the mechanisms underlying these differences may contribute to the development of therapies aimed at preventing disease onset or progression.

## Sex hormones in the brain

Prof. Kou Shinoda reviewed the actions of sex hormones in the brain and the regulation of their receptors through crosstalk among steroids [3–6]. Prof. Shinoda introduced the concept of a multidimensional spectrum of gender, emphasizing that human gender comprises diverse attributes, including: (1) social context such as legal registration and name; (2) cellular and molecular sex determined by genes and sex hormones; (3) cutaneous and musculoskeletal sex characteristics, including mammary glands, buttocks, external genitalia, and facial features; (4) individual gender expression, such as voice, facial expression, clothing, and preferences; and (5) gender affinity, sexual orientation, and gender identity. He explained that individual expression and gender identity are related to brain differentiation regulated by genes and sex steroids. In particular, sex steroids shape and activate selective neural circuits among sex-sensitive neurons by inducing basal excitation. Crosstalk among different steroids also contributes to the actions of steroid receptors. In the mammalian brain, sex steroid receptors are abundantly expressed, and numerous estrogen-synthesizing neurons expressing aromatase—which converts androgens to estrogens—are found in the medial preoptic area, hypothalamus, and amygdala, regions known to exhibit sexual dimorphism. Prof. Shinoda suggested that locally elevated estrogenic status not only regulates steroid levels and receptor expression and contributes to sexual differentiation, but that reduced activity of these estrogen-synthesizing neurons may be involved in dysfunction of regulatory systems in the hippocampus, amygdala, hypothalamus, and monoaminergic neurons, potentially contributing to dementia, autonomic dysfunction, or depression. Furthermore, membrane receptors for sex steroids have also been reported, indicating that the mechanisms underlying sexual differentiation of the brain are complex.

Recent studies have identified stigmoid bodies (STBs), which bind sex steroid receptors and regulate their nuclear translocation in steroid-responsive regions of the brain. Huntingtin-associated protein 1 (HAP1) is localized in STBs and may play a protective role in spinal and bulbar muscular atrophy (SBMA), which is caused by polyglutamine expansion in the androgen receptor, as well as in Huntington’s disease.

Prof. Shinoda concluded that the distribution, expression, nuclear translocation, and activation of sex steroid receptors, as well as their crosstalk, vary substantially across brain regions. He proposed a hypothesis regarding the roles of estrogen-synthesizing neurons in the brain and comprehensively discussed multiple aspects of steroid actions in the brain and the diversity of brain gender, as well as their potential contradictions with gender-divided society.

## Gender differences in social ability in twins

Prof. Chisato Hayashi presented her recent study on gender differences in genetic and environmental factors associated with social ability and school adaptation in twins [7].

The study found no significant gender differences in sociability. In contrast, significant qualitative and quantitative gender differences were identified in the genetic and environmental contributions to school adaptation. The effect of additive genetic factors was greater in males than in females, whereas the effect of non-shared environmental factors was greater in females than in males. These findings suggest that gender-sensitive approaches may be effective in supporting school adaptation. For example, consultation-based support such as counseling or assistance in identifying underlying problems may be beneficial for males, while interventions focusing on class changes or management of peer relationships may be more effective for females.

## Gender difference in Alzheimer’s disease

Prof. Gaku Ichihara presented a review on gender differences in Alzheimer’s disease (AD). Epidemiological studies consistently show that the morbidity of AD is higher in females than in males across all age groups [2, 8]. This finding contrasts with many other diseases, such as various cancers and cardiovascular diseases, which generally show higher morbidity in males. This unique pattern represents a notable singularity in gender differences in disease, and clarification of its mechanism may contribute not only to understanding AD pathogenesis but also to elucidating gender differences in other diseases. Available data indicate that: (1) the morbidity of AD is higher in females than in males throughout all ages, and (2) the gender difference becomes more pronounced after menopause. Prof. Ichihara emphasized that these two points should be discussed separately. First, the dual role of estrogen should be considered when interpreting gender differences in AD. Estrogen therapy reduces the risk of cognitive dysfunction when initiated soon after menopause or before the age of 65. In contrast, when estrogen therapy is started more than 10 years after menopause or after the age of 65, it becomes ineffective or may even increase the risk of cognitive dysfunction. These findings suggest that estrogen exerts dual effects depending on major endocrine changes across the female lifespan. Previous studies have also reported that the effects of estrogen therapy differ according to APOE polymorphisms. Prof. Ichihara noted that his group’s recent studies have demonstrated dual effects of Nrf2 [9], which is activated by estrogen. Therefore, it is necessary to clarify which genetic and external or internal environmental conditions determine whether estrogen is protective or harmful to the brain. Second, Prof. Ichihara discussed differences in estrogen receptor subtypes. Estrogen receptor α is mainly expressed in the mammary gland, whereas estrogen receptor β is abundantly expressed in the brain. Furthermore, gender differences have been reported in the expression of estrogen receptor β. Polymorphisms in estrogen receptor genes are known to be associated with increased AD risk, and this association appears to be stronger in females than in males. These findings indicate that investigation of gender differences in the brain itself is essential for clarifying the mechanisms underlying gender differences in AD. Third, testosterone may contribute to lower AD risk in males [10]. Testosterone has been reported to inhibit β-amyloid secretion and promote β-amyloid degradation, and previous studies suggest that testosterone therapy may reduce AD symptoms. These mechanisms may partly explain the higher risk of AD in females even before menopause. Fourth, Prof. Ichihara highlighted the potential contribution of gender differences in external and internal environmental factors that promote cognitive dysfunction. Recent epidemiological studies have reported an association between air pollution and cognitive decline and particularly higher susceptibility in young females with APOE4 allele and higher BMI. Prof. Ichihara concluded that further studies are needed to clarify the dual roles of estrogen and Nrf2, which is activated by estrogen, in AD and cognitive dysfunction.

## Discussion

The symposium highlighted that gender differences exist in susceptibility to neurodegenerative diseases; however, the more susceptible gender varies depending on the disease. Among neurodegenerative diseases, epidemiological evidence indicates that females are more susceptible to Alzheimer’s disease. Several factors should be considered to understand this gender difference, including the dual effects of estrogen depending on the timing of hormone therapy, the effects of testosterone, the influence of the APOE4 allele, and environmental factors. Furthermore, investigating the crosstalk between sex steroid hormone receptors in the brain is needed to clarify the biological mechanisms underlying gender differences in susceptibility to neurodegenerative diseases. Differences in social behavior and social functioning between sexes should also be considered as potential factors contributing to gender differences in the development and progression of neurodegenerative diseases.

Overall, the symposium emphasized the need for further research on the roles of estrogen and genetic factors to better understand the mechanisms underlying gender differences in neurodegenerative diseases and behavioral problems.

## Declarations

### Availability of data and materials

There are no new data associated with this article.
